# Rhythmic Structure Shapes Dyadic Self−Other Representations Through Interpersonal Action Coupling

**DOI:** 10.1111/nyas.70344

**Published:** 2026-08-03

**Authors:** Dhwani P. Sadaphal, Günther Koliander, Christian R. Blum, Jan Stupacher, W. Tecumseh Fitch, Peter E. Keller

**Affiliations:** ^1^ Department of Behavioral and Cognitive Biology University of Vienna Wien Austria; ^2^ Acoustics Research Institute Austrian Academy of Sciences Wien Austria; ^3^ Center for Music in the Brain, Department of Clinical Medicine Aarhus University Aarhus Denmark; ^4^ The MARCS Institute for Brain, Behaviour, and Development Western Sydney University Sydney New South Wales Australia

**Keywords:** interpersonal coordination, meter, polyrhythm, rhythm, self–other representations, task‐sharing

## Abstract

Moving in unison to a musical beat can blur boundaries between individual movements, fostering prosociality. However, structured patterns of sounds and silences in rhythms (meter) allow various ways of moving to the beat. How do these different rhythmic interpretations affect social connectedness? Unacquainted dyads drummed in unison, and at more complex integer multiple and polyrhythmic ratios. Coordination requires continuously tracking who does what and when. We varied trackability by manipulating task‐sharing (the same or separate drum pads, and audible or inaudible partner drumming). More complex ratios decreased social connectedness. However, coordination revealed a dichotomy between unison and the more complex ratios: in unison drumming, participants automatically tracked and adjusted to each other's actions, but both integer multiples and polyrhythms separated the two participants’ actions in time, allowing one to be stable despite their partner being variable. Hearing each other increased participants’ mutual reliance on each other's actions, but by itself did not increase connectedness. However, sharing a drum pad and audio significantly boosted self–other merging ratings. Notably, more stable coordination facilitated integrated self–other representations. Our findings are consistent with the premise that meter provides a shared cognitive‐motor scaffold that enables group cohesion, while simultaneously allowing individual expression through multi‐part rhythm production.

## Graphical abstract

Musical rhythms can imply multiple interpretations of the beat, allowing various ways of moving together. Dyads drummed to songs, producing rhythmic ratios varying the relative temporal complexity of their movements. Stable coordination increased social bonding, which decreased with complexity. Hearing the partner promoted tracking of their movements, but goal‐sharing in addition to hearing increased connectedness. The findings suggest a role of meter as a shared temporal schema for social bonding.

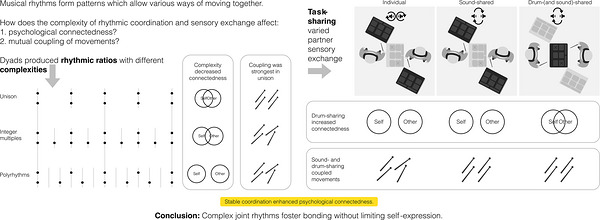

## 1 Introduction

Music features prominently in social gatherings around the world. It often inspires repetitive movements to a beat, such as singing, clapping, and/or dancing, while nonetheless allowing diverse forms of self‐expression. Music with a regular beat gets people moving in synchrony, and promotes affiliation and cooperation [1, 2, 3, 4]. When moving in unison with others, music acts as a common ground, structuring repetitive movements and predicting one's own and others’ actions. The resulting group activities can blur interpersonal boundaries, leading to social bonding, which manifests as increased cooperation, liking, and feelings of connectedness towards others [5, 6, 7, 8].

Music‐making is a communicative behavior that generates auditory signals at various rates, forming multiple rhythmic timescales. Listeners spontaneously organize these complex arrangements into a predictive hierarchy of sounds and accents, known as *meter* [9, 10, 11, 12, 13]. Meter affords several simultaneously valid ways to synchronize with music [14, 15]. Thus, human brains interpret rhythms in diverse manners [16, 17, 18, 19]. Musical ensembles expertly exploit this diversity to create captivating soundscapes that dynamically emphasize various rhythmic features. Owing to strong auditory−motor connections in the human brain, individual rhythmic movements can vary based on which rhythmic features capture attention [17, 19, 20, 21, 22, 23, 24, 25, 26]. The present study addressed such diversity in individual rhythmic performances, including and beyond unison, in a dyadic context. Specifically, we tested the effect of producing various joint rhythmic structures, across differing social contexts, on feelings of social connectedness and the variability of coordination.

In experienced musical ensembles, diverse rhythmic parts affect coordination by shaping internal models of each person's actions [21, 27, 28]. Internal models mentally represent expectations of who does what, and when, based on action‐contingent sensory feedback. Here, action–perception coupling plays an important role. Specifically, access to a duet partner's auditory feedback stabilizes one's own performance [29, 30]. Other sensory modalities, such as vision and proprioception, have similar contributions [31, 32, 33]. This has been considered evidence that musicians continuously track and simulate auditory outcomes of both their own and others’ actions [34]. These internal models help adjust one's movements to maintain a common goal of coordinating cohesively [8, 35, 36, 37, 38]. Therefore, actively engaging rhythmic tracking mechanisms, and integrating sensory consequences of dyadic or group actions, is a central feature of joint musical rhythm.

While trained musicians expertly navigate these challenging coordination demands, individuals without formal training (non‐musicians) also form cohesive self–other representations based on rhythm. Studies comparing synchrony via phase‐aligned joint isochronous (regular, equally spaced) actions with asynchronous (phase‐misaligned) actions have shown this consistently [1, 31, 39, 40, 41, 42]. Moving isochronously in unison does not require particularly skilled coordination or training, or even active engagement [11, 43], yet supports remarkably prosocial payoffs: joint rhythmic action yields phase‐alignment, spontaneously integrating self–other internal models. In contrast, tracking phase‐misaligned actions requires separate self–other internal models, and hence does not foster integration. Notably, these sensorimotor representations are socio‐cognitive [7, 38]. Simply tapping in unison with an inanimate device, such as a metronome, does not significantly promote affiliation toward a nearby human partner [1, 39, 44]. Psychological feelings of bonding arising from unison‐driven self–other integration are affected by individual empathic traits [20, 41, 45] and engage neurohormonal mechanisms [46, 47, 48] and neural areas associated with social cognition [49, 50].

Joint rhythms other than unison, with temporally segregated but structured individual actions, can be produced accurately despite increased challenges in tracking others’ actions [21, 51]. For instance, alternating rhythms can prove just as stable as unison, in comparison to other phase‐shifted arrangements [31, 32]. Meter facilitates such coordination. Imposing metrical hierarchies improves accuracy when performing challenging rhythmic tasks, such as taking turns to produce a melody, one note at a time [52]. Similarly, actions produced at mismatched rates can be integrated into meaningful representations by attentionally mediated top‐down modulation [38, 53]. An earlier study found that coordination was more stable for pitch‐segregated than pitch‐matched actions [54]. The authors proposed that distinct pitches aided coordination, in addition to the visual congruence from moving in unison, by helping distinguish self–other contributions. Therefore, using self–other segregation to one's advantage may be a key to success in demanding rhythmic tasks. According to a recent evolutionary account, the tracking and integration of temporally non‐overlapping, but structured, rhythmic contributions may signal social identity across complex, hierarchical social structures [55].

**FIGURE 1:**
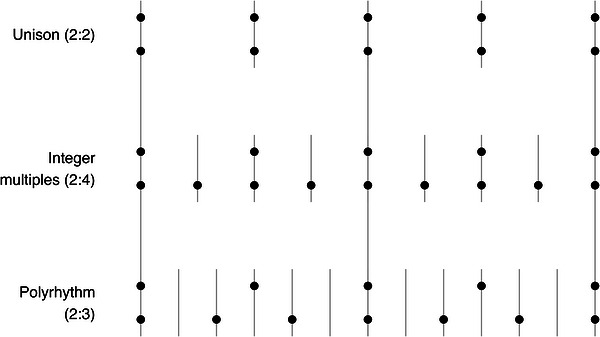
The complexity of multi‐part rhythms. Two bars (repeating rhythmic cycles) of different rhythmic ratio streams (time along the horizontal axis). Long vertical lines represent bar onsets, short vertical lines represent subdivision onsets. Two complex features are evident in the polyrhythm in comparison with the unison and integer multiple rhythms. First, the 2:3 polyrhythm is more complex than the others because it requires six subdivisions, as compared to four for 2:4 and two for 2:2. (The smallest temporal subdivision required to represent all beats in the two streams is derived from the least common denominator of the number of beats within each bar of the two streams.) Second, the polyrhythm groups the subdivisions heterogeneously (2‐1‐1‐2), which is more complex than 1‐1 for 2:2 and 1‐1‐1‐1 for 2:4.

Producing challenging multi‐part rhythms also benefits from social interaction. This was recently shown in a study of dyads drumming a 2:3 polyrhythm [56]. Polyrhythms consist of two (or more) concurrent rhythmic streams. At their most basic repeating pattern, any two polyrhythmic streams contain a coprime number of beats. Producing them, therefore, requires a complex temporal grid to be imposed cognitively (Figure 1) [24, 51, 57, 58]. Polyrhythmic drumming improved with background music, which provided a structural foundation for the rhythm, as did moving from virtual reality to an in‐person setting [56]. Another study found that rhythmic ratio complexity systematically affects judgments of social connectedness [59]. Online participants were asked to imagine being one of two animated avatars they viewed producing different rhythmic ratios together. Compared with the most complex 4:5 polyrhythm and unstructured rhythms with random inter‐beat intervals, structured ratios yielded higher self–other merging ratings. The decrease in self–other merging with increasing ratio complexity was taken as evidence that participants tracked and integrated their own and the others’ actions. Interestingly, rhythmic structures potentially influence whether an individual leads or follows [60], and when asked to freely coordinate, people build strong rapport by making novel and engaging movements, instead of predictable and repetitive ones [61]. In sum, these findings suggest a complex relationship between rhythmic structure, sensorimotor processes, and social connection. However, it remains unclear how self–other representations arise from metrically structured multi‐part rhythmic coordination, beyond 1:1 phase relationships.

Internal models of joint rhythmic action, based on spatio‐temporal relations between individual rhythmic components, are coupled together by tracking and integrating one's own and others’ action outcomes [5, 21]. The communication required for such monitoring, especially when producing difficult rhythms, benefits from task‐sharing, which entails a common task goal and heightened sensory exchange [62, 63, 64, 65]. Individuals mutually adapt when they can see and hear each other, and this reflects in measures of temporal stability [30, 66, 67, 68]. When playing particularly challenging, irregular rhythmic passages, musicians show heightened attention toward their own and others’ actions, making use of communicative gestures to stabilize performance [69]. Even in non‐musicians, inter‐brain synchrony increases with the stability of tapping joint alternating rhythms [70]. Taken together, rhythmic complexity and task‐sharing can influence the coupling strength of internal models, and hence overlap with self–other representations in the sensorimotor system.

The research presented here addressed two main questions. First, we asked whether rhythmic ratio complexity and task‐sharing shape self–other representations and affect coordination stability (Q1). Second, we asked whether individuals’ reliance on each other's actions (mutual coupling) is affected by rhythmic ratio complexity and task‐sharing (Q2). Testing this idea in the general population poses challenges, because jointly producing rhythmic ratios typically involves high skill levels and joint practice. We overcame this issue by using audiovisual aids and background music, enabling unaquainted dyads with no musical training to produce challenging coordinated rhythmic ratios.

We cued dyads to produce rhythmic ratios of three types: unison, integer multiples, and polyrhythms. Task‐sharing was manipulated using drum pads and audio routing. The three task‐sharing conditions were: “individual” (separate drum pads, participants heard only their own audio), “sound‐shared” (separate drum pads, but both participants heard both audio streams), and “drum‐shared” (shared drum pad, shared audio). We recorded drumming throughout the task, and collected trial‐wise Inclusion of the Other in the Self (IOS) [71, 72] ratings. IOS ratings reflect social connectedness on a continuum between segregated to integrated representations of self and other [71, 73, 74]. We used the standard deviation of asynchronies as a measure of drumming variability, inversely indexing coordination stability. We hypothesized that, when individuals produce temporally segregated rhythms, hierarchical rhythmic structure (meter) grounds sensorimotor internal models and socio‐cognitive representations, by acting as an attention‐allocation scheme supporting the tracking and integration of sensory outcomes of one's own and others’ actions. Therefore, coordination stability was expected to be higher with integer multiple rhythms than with polyrhythms.

To answer the question of whether ratio complexity and task‐sharing affect self–other representations (Q1), we analyzed whether IOS ratings were predicted by the rhythm type (indexed by the complexity of the rhythmic structure underlying the ratio), task sharing, and combined drumming variability. We expected more complex rhythms to be more difficult to track and integrate, leading to more segregated self–other representations. Thus, for integer multiples and polyrhythms, we predicted a facilitative effect of task‐sharing on self–other merging. We also expected more stable coordination (lower combined drumming variability) to result in more integrated self–other representations.

To test whether dyads’ mutual coupling is affected by ratio complexity and task‐sharing (Q2), we first addressed a possible confound in the task design, namely, that audiovisual cues were present throughout the task to accommodate non‐musicians, and both participants in the dyad thus experienced rhythmically congruent contexts. We were interested in mutual influence on drumming through information transfer in social interactions. However, the consistently congruent rhythmic cues included potential influences on drumming unrelated to the interaction. To account for these in our analyses, we additionally assigned pseudo‐partners for each participant, randomly mapping them to another participant from the same study. This allowed us to isolate the effects of mutual coupling resulting from the real dyadic interaction, while controlling for other sources of variability that remained present in pseudo‐partners. We expected that only the real partners’ drumming, and not the pseudo‐partners’, would predict mutual coupling patterns. Here, mutual coupling was operationalized as the effect of the partner's drumming variability on the responder's drumming variability. Coupling was predicted to weaken as rhythmic complexity increased. Due to the strong crosstalk between auditory‐motor internal models, we expected sound‐sharing to increase participants’ reliance on each other's movements, aiding coordination.

## 2 Materials and Methods

### 2.1 Participants

Participants (*n* = 50 dyads, eight exclusions) were recruited through social media channels and the research participation pool of the Centre for Music in the Brain, at Aarhus University, Denmark. The recruitment notice was also displayed (with permission) at student housing venues and other local establishments. Dyads were reshuffled if the two participants reported having known each other prior to performing the experiment. We recorded data from all participants provided they were at least 18 years old, fluent in English, and reported healthy hearing, vision (including corrected vision), and movement capabilities. During data collection, we employed a pre‐defined exclusion criterion to identify disengagement from the task, that is, sessions with one or both of the participants giving the same rating on more than 90% of the trials (*n* = 5 dyads) would be excluded. Other exclusions (*n* = 3 dyads) were due to the participants having issues understanding instructions in English and using the apparatus.

This resulted in a final sample size of 42 dyads, balanced across the between‐dyads factor of song, used as background music (seven dyads per song). Demographics in the sample were fairly diverse in age (M = 27.0 years, SD = 7.07 years), gender (55 female, 26 male, 3 diverse), and 37 countries of origin (52 European participants, 22 Asian, and 10 other). Of these, 24 dyads consisted of participants identifying as the same gender. Participants had a range of Goldsmiths Musical Sophistication Index (Gold‐MSI) [75] scores (M = 45.5, SD = 5.37), Brief Interpersonal Reactivity Index (B‐IRI) [76] perspective‐taking scores (M = 10.7, SD = 3.12), and spontaneous motor tempi (M = 106 bpm, SD = 53.4 bpm).

Data were collected from the 7th of January 2025 until the 28th of February 2025. All participants provided informed consent before performing the task and were paid for participation. The study was approved by the Institutional Review Board of the Department of Clinical Medicine—Center for Functionally Integrative Neuroscience, Aarhus University (case number: DNC‐IRB‐2024‐006).

### 2.2 Design

**FIGURE 2:**
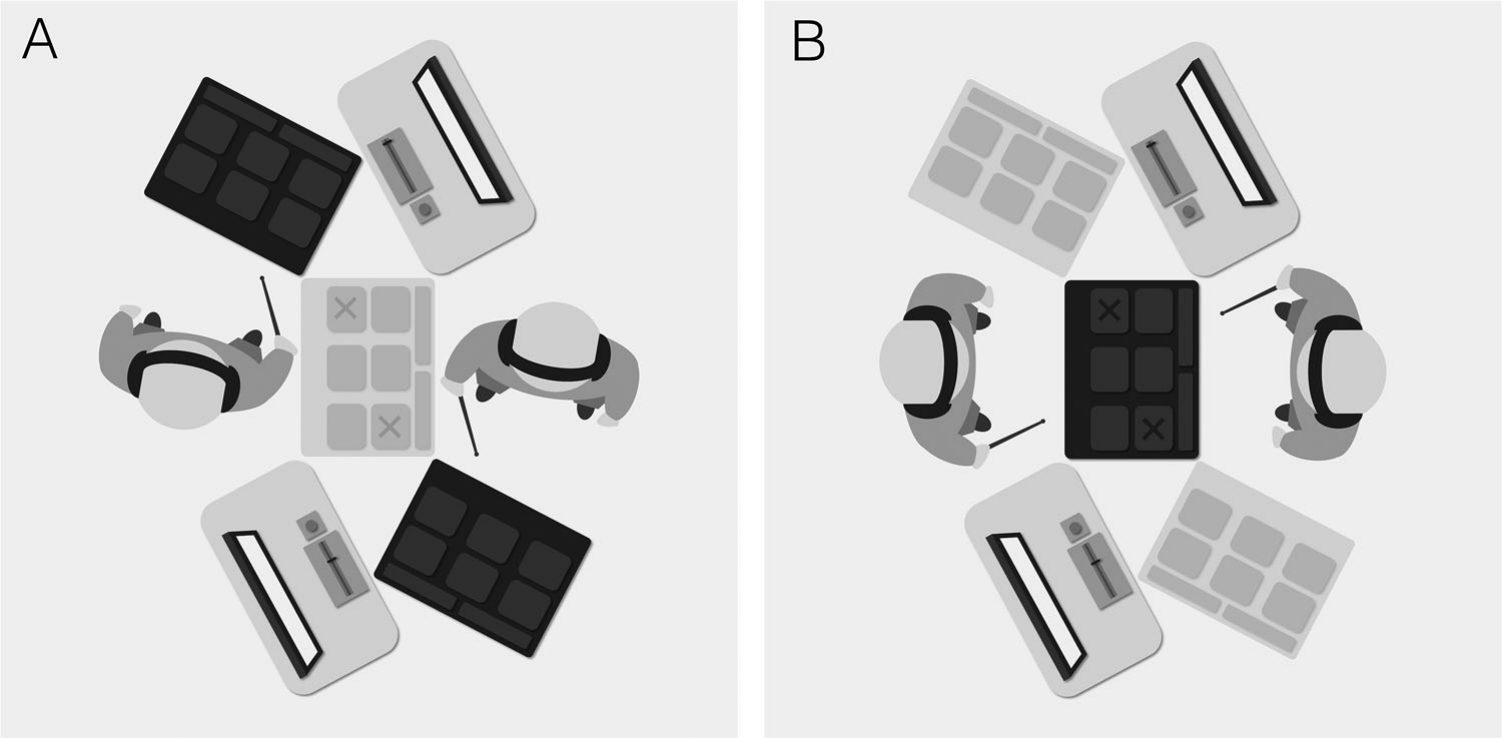
Drumming task setup. Participants had their individual MIDI drum pad to their left, the shared drum pad in between them, their own drumstick, headphones, monitor, and response devices (rating slider and push button). The setup was identical for both participants from an egocentric perspective. Participants oriented themselves toward (A) their individual drum pad in the “individual” and “sound‐shared” condition, and (B) the shared drum pad in the “drum‐shared” condition.

We implemented a full‐factorial within‐participants design by manipulating two key predictors: rhythm type and task‐sharing. A third test predictor in subsequent analyses was the combined drumming variability of the two participants, derived from the drumming data. Task‐sharing was manipulated at three levels: individual, sound‐shared, and drum‐shared (Figure 2). In the “individual” condition, participants used separate drum pads and could not hear each other's audio output. In the “sound‐shared” condition, audio from both drum pads was routed to both participants’ headphones, allowing them to clearly hear each other drumming. In the “drum‐shared” condition, participants used a shared drum pad and also heard each other drumming. It should be noted that participants could see each other in all three task‐sharing conditions.

**FIGURE 3:**
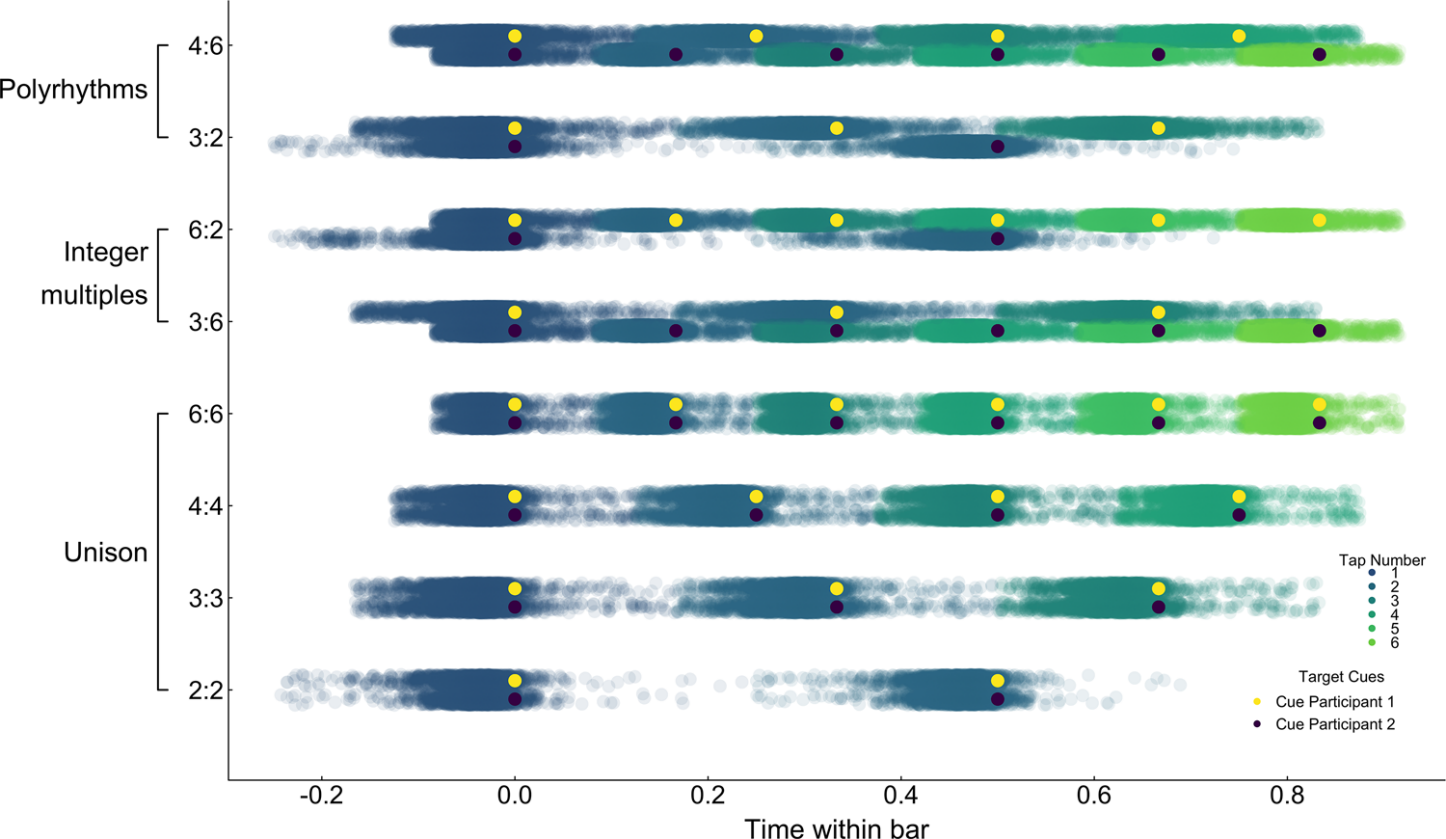
Target cues and participants’ taps. A plot of the normalized timing of the cued rhythmic ratios within each rhythm type against participants’ taps with superimposed target cues (metronomes and pacing video).

The rhythm type refers to the nature of the rhythmic ratio produced by the participants in a dyad: unison (2:2, 3:3, 4:4, and 6:6), integer multiples (3:6 and 6:2), and polyrhythms (3:2 and 4:6). The ratios within each rhythm type were based on the absolute number of taps in each bar (6‐beat cycle) corresponding to participant 1 and participant 2, respectively. The ratios 4:4, 6:6, and 4:6 were included to control for the effect of fast‐tempo drumming otherwise present only in integer multiple rhythms. Rhythmic ratios were cued by asking participants to drum along to audible metronome tracks and additional visual aids (Figure 3), set to match the instrumental background songs in a 6‐beat meter.

A note on the metrical context imposed by the background music: the songs often contained a bass drum on beat 1 and a snare on beat 4 of a 6‐beat metric cycle. Intuitively, this induces a swaying waltz‐like feeling (counted as “One‐and‐a, Two‐and‐a”). Thus, the 2 beats‐per‐bar grouping aligned more naturally with the music than others. On the other hand, the 3 beats per bar grouping feels march‐like (counted as “One‐and, Two‐and, Three‐and”). To account for these different metrical emphases imposed by the songs, metronomes, and clave sounds in the polyrhythm, we counterbalanced the rhythmic ratios across participants, such that both participants produced both metrical interpretations (waltz‐like or march‐like). Specifically, in the 4:6 ratio, participant 1 produced 4 beats‐per‐bar, while participant 2 produced 6 beats‐per‐bar. In the 3:2 ratio, participant 1 produced 3 beats‐per‐bar, while participant 2 produced 2 beats‐per‐bar. The possible effect of the different groupings was also controlled for in the analysis.

Due to the challenging coordination required in the task, we conducted a pilot study to assess the usefulness of auditory and visual cues, which revealed that visual aids were necessary because some musically untrained participants could not reliably perform the task in their absence. Song ID was a between‐dyads factor, analyzed as a random effect. Each dyad drummed to a single song, randomly selected from a set of six.

We recorded participants’ tap timing from MIDI drum pads throughout each trial, and their IOS [71, 72] ratings and enjoyment ratings on continuous 100‐point scales at the end of each trial as response variables. In addition to these trial‐wise responses, we recorded individual tasks and ratings, along with answers to standardized questionnaires (details in Section 2.4).

### 2.3 Stimuli

Percussive sounds (metronomes, drums; File S1) were prepared in Audacity v3.1.1.0 to last approximately 10 ms, normalized in loudness, and faded out to decay completely. The metronome track was constructed with a repeating bongo sound with a rich, woody timbre. Striking the MIDI drum pads triggered click‐like clave sounds with a sharp, cutting attack. Participant 2's clave could be distinguished from participant 1's by both pitch and timbre.

The six songs used as background music in the task were shortlisted from publicly available playlists of popular rock and pop songs. The tracks were stripped of vocals using the Python package spleeter v2.4.0. Then, 13‐bar tracks (File S2) were extracted, and their loudness was normalized in Audacity. Bar length ranged from 1.88 s for the fastest song to 2.21 s for the slowest. In other words, for a metrical interpretation of 6 beats per bar, tempi ranged from 162.87 to 191.49 bpm. We verified the expected bar lengths against perceived bar lengths in a pilot study with four participants who were asked to drum to all 6 beats in all song tracks. Expected bar lengths matched perceived bar lengths, validating them for further stimulus timing and analyses.

Visual pacing aids consisted of videos of a dancer displaying apparent biological motion, simulated by concatenating sequential still photographs (File S3). The movements followed a Rayleigh temporal profile, derived from openly available data from a previous study [77]. A red circle appeared in conjunction with the metronome pulse to visually cue the beat.

### 2.4 Procedure

Before arriving for the experiment, participants completed an online questionnaire with 4 items of the perspective‐taking subscale of the B‐IRI, 11 items of the short Gold‐MSI, all 7 items of the musical training subscale of the Gold‐MSI, and demographic information (age, gender, country of origin, and self‐reported handedness). Upon arrival, they provided informed consent and were individually escorted to the testing room to complete four initial tasks, in order: spontaneous motor tempo (two trials), free‐drumming with the song track with no cues (two trials), an initial IOS rating, and 10 trials of the Beat‐Drop Alignment Test (BDAT) [78]. The initial drumming tasks were performed on their respective individual drum pad.

The practice session introduced the song track, the metronome, the individual drum, the shared drum, and the dancing videos. Five practice trials with instructions (File S4) on the interpersonal drumming task preceded the main session, in order: one trial drumming on the individual drum pad, one trial drumming on the shared drum pad, followed by three trials also giving ratings introducing the three task‐sharing conditions (in order: individual, sound‐shared, and drum‐shared). The experimenter remained in the testing room during the practice session to help the participants with any issues. She left the room for the rest of the experiment. Practice trials were not analyzed.

This was followed by the main session, in which both participants performed the interpersonal drumming task together. Instructions at the beginning of each task‐sharing block indicated which drum pad (individual/shared) was to be used, and reminded them whether or not they would hear their partner's drumming. Each block contained a total of 24 trials: eight trials for each of the three rhythm types, balanced equally across ratios within each rhythm type. Task‐sharing block order was randomized across experimental sessions. Ratio order was randomized within each block, such that no two consecutive trials presented the same ratio. A brief LED flash and two beeps signaled that the trial was about to start. The session was self‐paced; participants were allowed to take breaks freely in between trials and resume the task by pressing the push button.

In each trial, participants heard the same 13 bars of the same song, with metronome tracks starting on the second bar, cueing their individual rhythm on that trial. Their task was to match the rhythm of the metronome, drumming using their dominant hand. They were instructed that the metronomes for the two people might sometimes be the same or different. To assist visually with synchronization, the monitors displayed an animation of a dancer whose movements were synchronized with the metronome. Participants were asked to watch the animation to pace themselves. Throughout the trial, we continuously recorded MIDI signal timing from the drum pads. At the end of each trial, participants rated their feeling of connectedness with the other participant on a continuous sliding 100‐point IOS rating scale. They also rated how enjoyable they found the trial on a continuous 100‐point scale (results not discussed).

At the end of the experiment, participants gave their final IOS ratings. These were not analyzed. During the entire experimental session, we also recorded participants’ individual and combined video footage (results not discussed). We ended each session with a debriefing interview in which we recorded whether participants recognized the song they encountered in the drumming task and their general comments or feedback, if any. Further details about the apparatus, stimulus presentation, and recording are available in the extended methods (File S5, including Supplementary Figure S1 therein).

### 2.5 Analysis

#### 2.5.1 Preprocessing

MIDI data were preprocessed in R v4.4.0 using R Studio scripts. Data were manipulated using the package *dplyr* v1.1.4. Raw MIDI note‐on events were filtered to only include data from the main task, excluding practice trials.

#### 2.5.2 Individual (Bar‐Wise) Drumming Variability

Taps occurring before the metronome track onset were removed. Bar timing was defined relative to the metronome track onset. Bars corresponding to the initial synchronization phase (bars 1–3) and the final bar (bar 13) were excluded from analysis, resulting in a total of nine analysis bars. These bars were excluded to focus the analyses on coordination once synchrony has been established, and to avoid potential missing data or changes in tap tempo (e.g., final slowing) associated with anticipating the end of the trial. Outlier inter‐tap intervals (ITIs) exceeding ±3 standard deviations from the participant's own trial‐wise mean ITIs were removed. Such extreme values reflect momentary breaks, possibly due to gaps in attention and motor execution errors. Additional taps within the same interval, triggered when striking the drum pads forcefully, were excluded. Tap timing was normalized by bar duration to yield relative timing measures. Asynchronies were calculated relative to the metronome cues, by subtracting the timing of the participant's tap from the metronome timing. Hence, negative asynchronies indicated early taps. The standard deviation of these asynchronies was calculated bar‐wise to reflect individual drumming variability.

#### 2.5.3 Combined (Trial‐Wise) Drumming Variability

To obtain one value of the two participants’ combined drumming variability in each trial, we first calculated interpersonal ITIs. We subtracted the target interpersonal ITIs from these to calculate asynchronies. In rhythmic ratios where the two participants’ taps were expected to overlap, we took participant 1's tap timing as the reference and subtracted participant 2's tap timing from it. We took the standard deviation of all these asynchronies across a trial to obtain the value of combined drumming variability.

Spontaneous motor tapping was quantified by calculating the median of the participants’ ITIs across the two trials. A single value of spontaneous motor tempo difference for each dyad was obtained by subtracting participant 1's median ITI from participant 2's median ITI.

#### 2.5.4 Inferential Statistics

Data were analyzed in R v4.4.0 with R Studio scripts, using generalized linear mixed‐effects models (GLMM) [79, 80]. We performed analyses at two levels of granularity: First, a trial‐wise analysis of the effect of the key test predictors rhythm type, task‐sharing, and participants’ combined drumming variability on IOS ratings (IOS ratings model‐Q1). Second, we conducted a bar‐wise analysis of the effect of one participant's drumming variability within the different rhythm types and task‐sharing blocks on the drumming variability of the other participant (interpersonal drumming model‐Q2). The following paragraphs outline the overall GLMM approach employed in both levels of analysis. Table S1 outlines the details regarding R packages, functions, and specific arguments. The fixed and random effects terms, results of diagnostic tests, and inferential test details for the two models are discussed separately in the extended methods (File S5, including Supplementary Figures S2‐S4 therein).

For both models, we performed diagnostics to check model stability and assumptions [81]. This included tests of overdispersion [82]. Variance inflation factors (VIFs) were assessed for a linear model with the main effects of all fixed effects’ terms, excluding any interactions, with no random effects. We checked collinearity issues by computing values of squared generalized VIF^1/(2×df)^ (henceforth “VIF”). Plots of best linear unbiased predictors were visually assessed for normal distributions and acceptable ranges [79, 83]. We used a custom function [84] to test overall model stability by refitting the model, eliminating each level within the random effects level one at a time, and comparing the resulting estimates to the full model. Additionally, we analyzed the influence of individual participants (for IOS ratings‐Q1) and individual dyads (for interpersonal drumming‐Q2) on the model's estimates using Cook's distance.

We started inferential testing with the comparison of a full model, reflecting the alternate hypothesis, to a null model, reflecting the null hypothesis [85]. This tests all relevant predictors together, preventing cryptic multiple testing [86]. To keep type I error rate at the nominal level of 0.05, we began with a maximal random effects structure with all identifiable random slopes [85, 87] based on data exploration using a custom function [84]. For the IOS ratings (Q1), we used a beta distribution with a logit link. For interpersonal drumming (Q2), we used a Gaussian distribution. If the maximal model did not converge, we eliminated random slope terms until we arrived at a full model that converged. We then performed the model diagnostics outlined above. The null model was identical to the full model, except that it did not contain the fixed effects terms concerning test predictors. This was followed by a Chi‐squared test of the full model against the null model, to test whether the full model explains the data better than the null model. If the *p*‐value of this comparison was significant, we continued with likelihood ratio tests [87] of individual fixed effects predictors to obtain *p*‐values for each fixed effects term. In the case of this model comparison being significant, but the test interaction revealing no significance, we followed up with likelihood ratio tests of individual fixed effects predictors of a reduced model including the main effects of the interaction, but lacking the interaction itself. Finally, we performed 1000 parametric bootstraps on the full model using a custom function [84] to obtain 95% confidence intervals for the key significant test predictors. Post‐hoc tests with *p*‐value adjustment [88] were done to conduct pairwise comparisons across levels of significant test predictors. We cite effect sizes in the extended methods (File S5) to encourage comparability across studies.

## 3 Results

### 3.1 IOS Ratings (Q1)

Self–other merging ratings were higher when dyads were more stable in their coordination, performed simpler rhythms, and shared both audio and a drum pad. The full model (Table 1) estimated IOS ratings as a function of the main effects of the test predictors, rhythm type, and task‐sharing, along with their interaction. An additional test predictor was log‐transformed combined drumming variability. Control predictors included the trial number, participant order, initial IOS rating, gender match, song recognition, score on the B‐IRI perspective‐taking subscale, spontaneous motor tempo difference, Gold‐MSI score, and the “ability” on the BDAT. Random effects were specified for participant and song, with random slopes of task‐sharing for both, and participant order for song. The null model differed from the full model only in the absence of the test predictors. The full‐null model comparison was significant (χ^2^ = 399.5, df = 27, *p* < 0.001), but the interaction of rhythm type with task‐sharing was not. The reduced model (Table S2) thus excluded the rhythm type–task‐sharing interaction, but included their main effects, and was otherwise identical to the full model. Likelihood ratio tests of individual test predictors in this reduced model yielded significant main effects of rhythm type (*p* < 0.001), task‐sharing (*p* < 0.05), and drumming variability (*p* < 0.001).

**TABLE 1:** Model summary (Q1) in link space for response variable IOS and the main effects and interaction of key test predictors rhythm type and task‐sharing, and the main effect of log‐transformed combined drumming variability.

Explanatory variables	Estimate	Standard error	*z*‐value	*p*‐value
Intercept	0.1424653	0.1464873	0.97	0.3308
Rhythm type Integer multiples^a^	−0.2161679	0.0439031	−4.9	8.50E‐07***
Rhythm type Polyrhythm^a^	−0.3186227	0.0435661	−7.31	2.60E‐13***
Task‐sharing Sound‐shared^a^	0.0594281	0.0752502	0.79	0.4297
Task‐sharing Drum‐shared^a^	0.3860248	0.1246009	3.1	0.0019**
Log Drumming variability^b^	−0.1757384	0.0149047	−11.79	2.00E‐16***
Trial number^b^	0.0478075	0.0225083	2.12	0.0337*
Participant order P2^a^	0.2399155	0.1429769	1.68	0.0933^+^
Initial IOS rating^b^	0.019945	0.0724046	0.28	0.783
B‐IRI Perspective‐taking^b^	0.0199138	0.071884	0.28	0.7818
Spontaneous motor tempo difference^b^	0.0024968	0.0729783	0.03	0.9727
BDAT Ability^b^	0.0161846	0.0751913	0.22	0.8296
Gold‐MSI score^b^	0.0248334	0.0748198	0.33	0.74
Gender match True^a^	0.0226392	0.1439629	0.16	0.875
Song recognition True^a^	−0.0745802	0.1676309	−0.44	0.6564
Integer multiples: Sound‐shared	0.031757	0.0620162	0.51	0.6086
Polyrhythm: Drum‐shared	−0.0371294	0.0617613	−0.6	0.5477
Integer multiples: Sound‐shared	−0.0000338	0.0623914	0	0.9996
Polyrhythm: Drum‐shared	−0.0244668	0.0627708	−0.39	0.6967

^a^ Reference levels: “Unison” for rhythm type, “Individual” for task‐sharing, “P1” for participant order, “False” for gender match, “False” for song recognition.

^b^ *z*‐transformed to an approximate mean of zero and SD of 1.

^+^ *p* < 0.1, *p < 0.05, **p < 0.01, ***p < 0.001.

**FIGURE 4:**
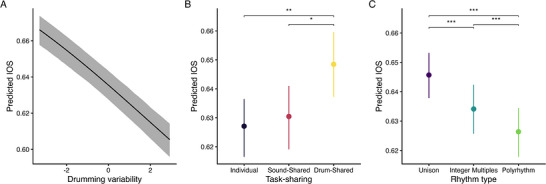
Bootstrapped model fitted estimates (Q1) of IOS ratings and 95% confidence intervals for the main effect of (A) log‐transformed combined drumming variability (*p* < 0.001; *x*‐axis depicts *z*‐transformed values), (B) task‐sharing (*p* < 0.05), and (C) rhythm type (*p* < 0.001), conditional on the effects of all other covariates and factors in the model being zero. *p < 0.05, **p < 0.01, ***p < 0.001. Abbreviation: IOS, Inclusion of the Other in the Self.

Lower drumming variability, indicating better coordination, predicted higher IOS ratings (Figure 4A). The control predictor “trial number” was also significant (*p* < 0.001; Figure S5), showing a predicted increase in IOS ratings over time. Post hoc tests showed that drum‐sharing was associated with significantly higher IOS estimates than individual and sound‐sharing conditions alone (Figure 4B; post hoc log‐odds ratios: sound‐shared vs. drum‐shared e = 0.726, se = 0.0974, *p* < 0.05; individual vs. drum‐shared e = 0.685, se = 0.0817, *p* < 0.01), but there was no difference between the latter two (post hoc individual vs. sound‐shared e = 0.944, se = 0.0625, *p* = 0.6608). Post hoc comparisons also revealed a significant difference between all three rhythm types, with estimates of IOS ratings declining as rhythms became more complex (Figure 4C; post hoc log‐odds ratios: unison vs. integer multiples e = 1.23, se = 0.0315, *p* < 0.0001; unison vs. polyrhythm e = 1.40, se = 0.0365, *p* < 0.0001; integer multiples vs. polyrhythm e = 1.14, se = 0.0307, *p* < 0.0001). Figure S6 shows a plot of the non‐significant test interaction of rhythm type and task‐sharing. Random effects were unremarkable (Table S3).

In an additional exploratory analysis, suggested by a reviewer, we tested whether the combined drumming variability mediates the effects of the rhythm type and task‐sharing on IOS ratings. This was done by including a three‐way interaction of these three variables in our main model. We compared this with a reduced model, which lacked the three‐way interaction term but retained the rest of this model's structure. A Chi‐squared test of these two models yielded a non‐significant *p*‐value (*p* = 0.752).

### 3.2 Interpersonal Drumming (Q2)

Participants’ drumming variability tracked their real partner's variability, showing different patterns across rhythm types and task‐sharing conditions. Mutual coupling was strongest in unison rhythms, and more pronounced with sound‐ and drum‐sharing. The full model (Table 2) estimated one participant's drumming variability (relative to the target cues) as a function of partner variability, its interaction with the rhythm type, and its interaction with task‐sharing. As control predictors, the model included the main effect of pseudo‐partner variability, its interaction with rhythm type, its interaction with task‐sharing, the trial number, bar number, and participant order. Random effects were specified for dyad, participant, song, and ratio. Random slopes included rhythm type, task‐sharing, and participant order within dyad and song; task‐sharing and participant order within ratio; and rhythm type and task‐sharing within participant. The null model excluded the test predictors but otherwise retained the structure of the full model. The full‐null comparison was significant (χ^2^ = 133.3, df = 40, *p* < 0.001), indicating that partner variability and its interactions explained response variability significantly better than pseudo‐partner variability, its interactions, and other controls. This allowed us to continue with likelihood ratio tests of individual test predictors, obtaining *p*‐values for the two test interaction terms. Both interaction terms of partner variability with rhythm type (*p* < 0.001) and partner variability with task‐sharing (*p* < 0.05) were significant.

**TABLE 2:** Model summary (Q2) for the logarithm of response variability and the interaction of key test predictors rhythm type and task‐sharing with log‐transformed partner variability.

Explanatory variables	Estimate	Standard error	*z*‐value	*p*‐value
Intercept	−0.03134	0.0792	−0.4	0.6923
Log Partner variability^a^	0.066	0.00942	7	2.50E‐12***
Task‐sharing Sound‐shared^b^	−0.0134	0.01628	−0.82	0.4107
Task‐sharing Drum‐shared^b^	0.00364	0.02864	0.13	0.899
Rhythm type Integer multiples^b^	0.00673	0.0849	0.08	0.9368
Rhythm type Polyrhythm^b^	0.26146	0.08497	3.08	0.0021**
Log Pseudo‐partner variability^a^	0.013	0.00936	1.39	0.1646
Trial number^a^	−0.00462	0.00621	−0.74	0.4565
Bar number^a^	0.00472	0.00385	1.23	0.2198
Participant order^b^	−0.06818	0.09493	−0.72	0.4727
Log Partner variability: Sound‐shared	0.02639	0.01005	2.63	0.0087**
Log Partner variability: Drum‐shared	0.02477	0.01013	2.45	0.0145*
Log Partner variability: Integer multiples	−0.08039	0.01062	−7.57	3.60E‐14***
Log Partner variability: Polyrhythm	−0.07388	0.0105	−7.04	1.90E‐12***
Log Pseudo‐partner variability: Sound‐shared	0.00536	0.00998	0.54	0.5916
Log Pseudo‐partner variability: Drum‐shared	−0.00323	0.01007	−0.32	0.7483
Log Pseudo‐partner variability: Integer multiples	−0.01999	0.01049	−1.91	0.0566^+^
Log Pseudo‐partner variability: Polyrhythm	−0.01794	0.0104	−1.72	0.0845^+^

^a^ *z*‐transformed to an approximate mean of zero and SD of 1.

^b^ Reference levels: “Unison” for rhythm type, “Individual” for task‐sharing, “P1” for participant order.

^+^ *p* < 0.1, *p < 0.05, **p < 0.01, ***p < 0.001.

**FIGURE 5:**
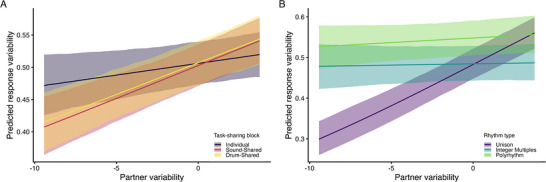
Bootstrapped model fitted estimates (Q2) of response variability (lines) and 95% confidence intervals (shaded bands) for the interaction of log‐transformed partner variability with (A) task‐sharing (*p* < 0.001), and (B) rhythm type (*p* < 0.001), conditional on the effects of all other covariates and factors in the model being zero. The *x*‐axis depicts *z*‐transformed values.

Post hoc tests showed that increases in partner variability predicted increases in response variability in both sound‐ and drum‐sharing; these did not differ from each other (Figure 5A; post hoc: sound‐shared vs. drum‐shared e = 0.00163, se = 0.0102, *p* = 0.8734). Additionally, both sound and drum‐sharing predicted response variability more strongly than the individual drumming (Figure 5A; post hoc: individual vs. sound‐shared e = −0.02639, se = 0.0101, *p* < 0.05; individual vs. drum‐shared e = −0.02476, se = 0.0101, *p* < 0.05). As depicted in Figure 5B, unison rhythms displayed the strongest coupling between partner and response variability (post hoc unison vs. integer multiples e = 0.08039, se = 0.0106, *p* < 0.0001; unison vs. polyrhythm e = 0.07388, se = 0.0105, *p* < 0.0001). Response variability in unison increased as partner variability increased. Both integer multiples and polyrhythms were significantly weakly coupled in comparison, and did not differ from each other (post hoc integer multiples vs. polyrhythm e = −0.00651, se = 0.106, *p* = 0.5407). There were no distinctive random effects variances, except for the random slope of participant order within ratio (Table S4), which may be explained by between‐participant rate asymmetry in the chosen ratios (e.g., 3:2 and 4:6, instead of 2:3 and 4:6).

An additional exploratory model (Table S5) contained a three‐way test interaction of partner variability with rhythm type and task‐sharing with simplified random effects. This model also included a three‐way interaction of pseudo‐partner variability with rhythm type and task‐sharing, along with all other control predictors, and a random effect of dyad. Likelihood ratio tests of individual test predictors in this model also yielded a significant *p*‐value only for the three‐way test interaction (*p* < 0.005), and not the pseudo‐partner interaction term. As shown in Figure S7, response variability consistently increased with partner variability for unison rhythms across all task‐sharing conditions. For integer multiples and polyrhythms, however, this relationship showed considerable variation across task‐sharing conditions. For integer multiples, higher partner variability led to an apparent decrease in response variability in the individual and drum‐sharing conditions, but the relationship was reversed with sound‐sharing. The trends were less pronounced for polyrhythms, with an inverse partner−response variability relationship in the individual and sound‐sharing conditions, and a modest but direct relationship with drum‐sharing.

## 4 Discussion

Our study investigated self–other representations and coordination patterns when individuals produce diverse rhythmic performances in the form of two‐part rhythmic ratios. Unacquainted dyads followed audiovisual cues set to popular songs, producing rhythmic ratios including unison and more complex integer multiples and polyrhythms, and provided trial‐wise self–other merging ratings. Task‐sharing manipulations using drum pads and audio routing revealed patterns in mutual action tracking when producing these challenging rhythms. The results suggest that jointly producing rhythmic ratios of varying complexity influences self–other representations across different task‐sharing contexts. Furthermore, the mechanisms that influence self–other representations during the production of these rhythms possibly overlap with the coupling strength of sensorimotor internal models. Overall, the study provides important insights about how social bonding emerges from performing metrically structured multi‐part rhythms, beyond simple 1:1 phase relationships.

Our two research questions concerned the subjective merging of self–other representations (Q1), and the mutual coupling patterns in the dyads’ drumming (Q2). We found that IOS ratings, reflecting self–other merging, differed systematically across rhythm types (specifically, the complexity of the rhythmic structure underlying the ratio), task‐sharing, and drumming variability. Additionally, participants’ mutual reliance on each other's actions implied different coupling strengths across rhythm types and task‐sharing conditions, while analytically controlling for factors unrelated to the interaction, that is, congruent rhythmic cues to both participants in the dyad.

The self–other merging (Q1) results largely aligned with predictions from our hypotheses, and with earlier research. Increasingly complex coordination received significantly lower IOS ratings, consistent with the hypothesis that the sensory segregation of one's own and others’ actions systematically influences self–other merging. This pattern was also found for rhythmic ratio complexity in our previous online study [59], and in earlier studies employing phase‐alignment manipulations [1, 31, 39, 40, 41, 42]. We expected sound‐ and/or drum‐sharing to facilitate self–other integration; however, sharing audio and a drum pad led to significantly higher ratings than the other two conditions. This suggests that sharing the drum pad, in addition to audio, emphasized social aspects of the interaction, yielding stronger feelings of connectedness. Specifically, drum‐sharing induced an unprompted feeling of shared goals, which are, in turn, known to enhance the prosocial effects of synchrony [29, 89, 90]. However, a predicted interaction between rhythm type and task‐sharing was not significant. The significant main effects of rhythm type and task‐sharing, therefore, suggest a role of higher cognitive interpretations on self–other merging. IOS ratings increased with trial number, which we attribute to an increasing feeling of social connectedness as more time was spent with the partner.

A notable finding regarding the IOS ratings (Q1) was a link between the combined drumming variability and self–other merging. Consistent coordination, operationalized as lower variability of interpersonal tap intervals, was accompanied by higher IOS ratings. This supports the hypothesis that individuals are sensitive to their collective performance, and that this influences self–other representations. The interpersonal drumming (Q2) results strengthened this interpretation. Self–other merging was not merely an emergent phenomenon unrelated to the social interaction. Indeed, we found a robust effect of the real partner's drumming variability, along with its interactions with rhythm type and task‐sharing, on response variability. The same analysis showed no effect of a pseudo‐partner's drumming, nor its interactions with these predictors, indicating that the observed coupling of participants’ drumming variability was not an artifact of the congruent rhythmic cues presented to both participants in the dyad.

Interestingly, there was some disparity in how participants represented each other cognitively and how much they adapted to each other's actions. IOS ratings (Q1) did not differ across individual drumming and sound‐sharing, and were significantly higher only for drum‐sharing. This contradicted our prediction that auditory exchange (in sound‐sharing) alone suffices to integrate self–other representations by encouraging coordination with the partner. However, participants tracked each other's actions (Q2) equally strongly with sound‐ and drum‐sharing, compared to drumming on separate drum pads with no auditory exchange (individual drumming). The two participants’ individual variability was directly related to sound‐ and drum‐sharing (but not individual drumming), compatible with the hypothesis that individuals track action‐contingent sensory feedback from each other's actions to coordinate various rhythms. This aligns with earlier studies using similar auditory routing manipulations [29, 39, 56, 65, 91]. Together, these results suggest that the collaborative feeling of a shared goal may hold considerable socio‐cognitive weight in representing the self and others, and attenuates the contributions of mutual action‐adaption as sensory exchange increases.

The effects of rhythm type also differed among cognitive representations and mutual adaptation patterns. While IOS ratings (Q1) significantly differed across all rhythm types, our results revealed a dichotomy between unison and the other more complex rhythmic ratios on individual drumming variability (Q2). In unison, participants’ drumming became more susceptible to variation in the other's drumming. Coupling in this case was apparently automatic, suggesting that participants could not, or did not, separate the self from the other during identical action. This automatic coupling was not observed for integer multiples and polyrhythms, although they were overall more variable due to their complexity. Participants’ variability producing these more complex rhythms was, in fact, affected less by more variation in the partner. This supports the hypothesis that more complex temporally segregated rhythms facilitate clear tracking of, and distinction between, one's own and others’ actions. Overall, unison drumming seemed to compel the dyad to function as one [92], boosting the feeling that the group is unified and cohesive, but more complex rhythms emphasized individuality while maintaining cohesion, despite requiring more difficult parsing. An alternative explanation could be that participants simply ignored each other while producing these more complex ratios. Interestingly, IOS ratings for various rhythmic ratios from an earlier online study resembled the mutual action coupling patterns (Q2) more than the cognitive self–other representations (Q1) in this study [59]. This may have been because this previous study involved no real movement or social interaction, making integrated cognitive representations mainly reliant on audiovisual tracking.

Several limitations constrain the broader interpretation of the results. First, although our analyses addressed a potential confound of consistent rhythmic cues, the design was not suited to observing unfolding patterns of leading and following that might emerge when participants continue drumming without cues. Such patterns may be highly informative about dynamic social roles in real‐world rhythmic coordination. Second, we used the standard deviation of asynchronies as a measure of performance. This is a well‐accepted measure of drumming variability [30, 68, 93, 94, 95, 96], and systematic differences between task‐sharing conditions in our analyses validate its use as a measure of mutual coupling. However, the ratios of integer multiples and polyrhythms in our study inevitably resulted in an unequal number of data points in calculating this measure for either participant. Furthermore, it may be argued that asking participants to rate interpersonal connectedness primed them to feel connected by framing the task in a social context. Nevertheless, this does not invalidate analyzing differences among these ratings to evaluate self–other representations in our task. In fact, some participants reported finding the exercise of repeatedly providing IOS ratings rather artificial. Finally, our choice of counterbalancing rhythmic ratio directionality between participants in a dyad was potentially suboptimal, because even though all participants performed both possible polyrhythmic beat groupings, they did not perform both slow and fast drumming rates. This was necessary to keep the experiment duration within reasonable time limits in our full‐factorial design.

With respect to the task‐sharing setup, we intentionally did not include a drum‐sharing condition with only self‐audio, since our hypotheses did not concern coordination in the absence of sound, but in the presence of other sensory inputs. Similarly, we did not visually obscure participants from each other using screens in the individual drumming condition. Our participants could arguably coordinate using peripheral visual inputs. We excluded these possible manipulations (“shared drum pad without shared audio” and “individual drumming without visual exchange”) both in the interest of brevity, and because it did not directly concern the study's aims. Moreover, our design maintained other task‐sharing aspects consistent between individual drumming and sound‐sharing, functioning as a robust test of auditory exchange, which was a variable of interest in our hypotheses. Finally, the novelty of rhythmic ratio variation is somewhat offset by the repetitive use of the same background music in the entire session.

Taking a step back, we find it remarkable that the results came from unacquainted participants with no musical training requirements, who successfully performed a quite challenging rhythmic task. Participants were neither instructed to collaborate and track each other's actions, nor told to notice how difficult it was to produce the complex rhythmic ratios. Despite this, results indicated that they perceived their combined drumming variability as a marker of social connectedness. This has been observed in other task domains [97, 98, 99, 100], and now in our musical rhythmic task. Furthermore, although the coupling of participants’ movements (Q2) did not differ for integer multiples and polyrhythms, subjective feelings of self–other merging (Q1) did scale with their complexity. This difference between cognitive representations and mutual coupling is noteworthy, and suggests that our participants cognitively differentiated between the structure of integer multiples and polyrhythms, despite both having a similar effect on their coordination. In other words, musicians and non‐musicians alike can implicitly interpret differences in the temporal structure of coordination to signal different levels of social bonding.

Our findings have further implications regarding musical rhythm as a framework for balancing novelty and repetition, and its use for self‐expression when individuals coordinate in groups. The complex relationship between meter, interpersonal coupling, and social interaction revealed in our study speaks to a broader role of rhythm in social bonding [55]. Hierarchical metrical structures seem to be relevant not only for low‐level effects on coordination through sensorimotor coupling, but also seem to affect higher cognitive processes, leading to enhanced social connection. Self–other representations in temporally segregated, yet metrically structured, rhythmic coordination might stem from intertwined socio‐cognitive and sensorimotor mechanisms. Previous studies with duetting pianists indicate that auditory−motor connections in the brain facilitate successful coordination via action simulation [21, 36, 37, 50, 63, 101]. These neural representations emerge spontaneously while coordinating, and dynamically track self–other actions [102, 103]. Individuals may still find it rewarding to modulate sensorimotor internal models by producing metrically complex rhythms, even at the cost of requiring greater cognitive effort. Large ensembles successfully perform challenging multi‐part music by employing rehearsal strategies involving a conductor whose movements can serve as memory cues [38]. Because our study was limited to dyads, extrapolation of our research to ensemble performances (or to groups with a conductor) is a topic for future research. However, we predict that our result of increased feelings of social bonding with accurate synchronization and joint task engagement will generalize to groups of more than two performers. The sensorimotor advantage of separating sounds in time [54, 104], while still fitting them within a shared metrical hierarchy, thus lends itself to signaling social commitment [105, 106], despite the cognitive cost of tracking individual group members’ movements.

In conclusion, our results showed that sensorimotor coupling in dyads producing rhythmic ratios of varying complexity is affected by their hierarchical temporal structure (meter), and that this impacts self–other representations. We demonstrated that temporal precision, modulated by the coupling of interpersonal actions, partly predicts the degree to which socio‐cognitive representations of oneself and others are merged. A supplementary finding revealed that goal‐sharing selectively enhanced self–other integration, regardless of interpersonal action coupling. The results generalized across dyads and individual participants, and withstood control analyses excluding alternative interpretations. Stated differently, social bonding can result from rhythmically complex coordination, and relies partially on the extent to which individuals track and mentally represent others’ actions in relation to their own, alongside socially relevant factors such as a feeling of shared goals. Our findings point towards a complex and dynamic interplay between rhythmic structure, interpersonal movement, and social bonding, and are consistent with the hypothesis that complex metrical rhythms can play a role in social bonding, while still enabling self‐expression during coordinated movement [55]. Our study unveils intricate interactions between sensorimotor and higher‐order socio‐cognitive processes in self–other merging during group music making. Future studies may investigate the limits of this balancing act between bottom‐up and top‐down processes, along with their neural underpinnings.

## Author Contributions

D.P.S.: Conceptualization, project administration, software, investigation, data curation, methodology, formal analysis, validation, visualization, writing – original draft, writing – review and editing; G.K.: Methodology, formal analysis, validation, writing – review and editing; C.R.B.: Methodology, formal analysis, validation, writing – review and editing; J.S.: Conceptualization, methodology, resources, writing – review and editing; W.T.F.: Funding acquisition, supervision, writing – review and editing; P.E.K.: Conceptualization, methodology, resources, supervision, writing – review and editing. All authors gave final approval for publication and agreed to be held accountable for the work performed therein.

## Funding

D.P.S., C.R.B., and W.T.F. were funded by the Austrian Science Fund (FWF) under the project DK Cognition and Communication 2 W1262 B29 Grant DOI 10.55776/W1262. Center for Music in the Brain is funded by The Lundbeck Foundation (R469‐2024‐1573) and Købmand Herman Sallings Fond.

## Conflicts of Interest

All authors declare they have no conflicts of interest.

## Supporting information



Supplementary Information: nyas70344‐sup‐0001‐File_S1_percussive_sounds.zip.

Supplementary Information: nyas70344‐sup‐0002‐File_S2_songs.zip.

Supplementary Information: nyas70344‐sup‐0003‐File_S3_videos.zip.

Supplementary Information: nyas70344‐sup‐0004‐File_S4_instructions.pdf.

Supplementary Information: nyas70344‐sup‐0005‐File_S5_extended‐methods.pdf.

Supplementary Information: nyas70344‐sup‐0006‐Figures_S5‐S7.pdf.

Supplementary Information: nyas70344‐sup‐0007‐Tables_S1‐S5.pdf.
