# Supplementary material for: Rhythmic Structure Shapes Dyadic Self−Other Representations Through Interpersonal Action Coupling
Source: Ann N Y Acad Sci. 2026 Aug 3;1562(1):e70344. doi: 10.1111/nyas.70344 (PMC13432789; doi:10.1111/nyas.70344)
Supplement: Supplementary file 4 — Supplementary Information: nyas70344‐sup‐0004‐File_S4_instructions.pdf. [file NYAS-1562-0-s003.pdf]

Setup instructions

"Welcome to the main session of our study about music in social interactions.

Please press the button to proceed."

"The drumpad placed on your left side is your PERSONAL drumpad. Please play your personal drumpad with your drumstick now, to acquaint yourself with your drum's sound.

Once you are done, please press the button to proceed."

"The drumpad placed in between you and your partner is the SHARED drumpad.

When you use the shared drumpad, please use only the pad marked on the TOP RIGHT corner. On the shared drumpad, you will always hear your own drum, and also your partner's drum.

Please play the shared drumpad to acquaint yourself with both yours and your partner's drum sounds.

Once you are done, please press the button to proceed."

Drumming instructions

"The experiment consists of several trials in which you will create some rhythms together. In every trial, you will both hear music in your headphones. Along with the music, you will hear an additional drum track. Your task is to MATCH THE RHYTHM OF THIS DRUM TRACK, as evenly as possible.

Please press the button to hear an example of these audio tracks."

"In each trial, there will also be a person dancing on the screen. This person's dancing is synchronised with the drum track. Please watch the screen to help keep your pace.

The tempo of the drum track might be different across trials. The drum track you and your partner hear might sometimes be the same or different. On some trials, you may hear your partner even if you are using your personal drumpad.

Please remember, your task is ALWAYS to match the rhythm of the drum track.

Please press the button to proceed."

Practice instructions

"Next, you will perform a practice trial on your PERSONAL drumpad. Please press the button to proceed."

“Next you will perform a practice trial on the SHARED drumpad. Please press the button to proceed.”

“At the end of each trial, you will be asked to answer some questions about your experience regarding that trial. You can adjust your response rating using the slider. Once you are sure of your rating, please press the button to submit it.
Please try to use the whole rating scale, including the middle and the extremes.

Next you will perform a practice trial on your PERSONAL drumpad followed by answering the questions. Please press the button to proceed.”

“Next you will perform a practice trial on the SHARED drumpad followed by answering the questions. Please press the button to proceed.”

“Next you will perform a practice trial on your PERSONAL drumpad, but you will still hear your partner. Please press the button to proceed.

Initial task instructions

“Welcome to the study.

Now you will perform 2 short rhythmic tasks of 2 trials each. Each trial lasts about 20 to 30 seconds. Please read the instructions for each task carefully.

Please press the button to proceed.”

Spontaneous motor tempo task

“In the following trials, your task is to drum evenly at a pace that feels most comfortable and natural to you. Drumming "evenly" means regularly like the ticking of a clock, or the sound of footsteps when walking. Please start drumming after the beep.

Please press the button to proceed.”

Free drumming

“In the following trials, your task is to drum along when you feel the beat of the music. Please start drumming when the song starts.

Please press the button to proceed.”

IOS rating instruction

“The following question is about how connected you currently feel to the other participant whom you just met.

Connectedness refers to how socially bonded or affiliated you feel in psychological (not physical) terms.

To respond, please drag the slider, which will move two circles labelled "self" and "other" closer together or further apart. Circles that are closer together indicate that you feel more connected to the other participant.

The connectedness ranges from completely separate (not connected at all) to completely overlapping (strongly connected).

To submit your response, please press the button. Press the button to proceed."

Block instructions

"For the following trials, please use your PERSONAL drumpad. You will ONLY HEAR YOURSELF drumming. Press the button to begin the block."

"For the following trials, please use the SHARED drumpad. You will be able to hear each other drumming. Press the button to begin the block."

"For the following trials, please use your PERSONAL drumpad, but you will be able to HEAR EACH OTHER drumming. Press the button to begin the block."
