# Supplementary material for: Rhythmic Structure Shapes Dyadic Self−Other Representations Through Interpersonal Action Coupling
Source: Ann N Y Acad Sci. 2026 Aug 3;1562(1):e70344. doi: 10.1111/nyas.70344 (PMC13432789; doi:10.1111/nyas.70344)
Supplement: Supplementary file 5 — Supplementary Information: nyas70344‐sup‐0005‐File_S5_extended‐methods.pdf. [file NYAS-1562-0-s004.pdf]

Apparatus

The main computer, a Macbook M1 Pro operating on MacOS Sonoma 14.6.1, controlled all devices in the setup. Three Alesis SamplePad Pro MIDI drum pads were connected to the main computer via a MOTU Micro Express MIDI USB interface. The drum pads were arranged in the experiment room and mounted on drum stands. Crosses marked the standing position for both participants. The “individual” drum pads were placed on either participant’s left side (Figure 2). The “shared” drum pad was placed in between the individual drum pads. This allowed them access to both the individual and shared drum pad without having to change their position, keeping interpersonal distance constant through all conditions. The shared drum pad was additionally marked to indicate which specific rubber pad each participant was supposed to use.

Both participants were provided with one drumstick and a pair of beyerdynamic DT 770 headphones. The headphones were connected to the main computer’s audio output port using a stereo y-splitter. All pads on the individual drum triggered a stereo clave sound. On the shared drum only the two marked pads triggered sounds, but triggers from the other pads were also recorded.

Instructions, stimuli, and questions were presented on monitors placed in front of the participants. Rating responses were collected using 10kΩ ± 20% 0.25W linear slide potentiometers whose position was represented in real time on a virtual rating scale on the monitors. Both rating scales were set to zero at the start of every experimental session. Participants submitted their response by pressing a momentary push button. The push buttons triggered 150 ms tones with distinct pitches corresponding to each participant’s button. Additionally, a 300 ms trigger was sent to three 5mm 635 nm (red) LEDs and a 0.1W, 8Ω piezoelectric speaker to indicate the beginning of every trial. The response devices, the LED and the speaker were interfaced with the main computer through an Arduino Uno Rev 3 (Figure S1).

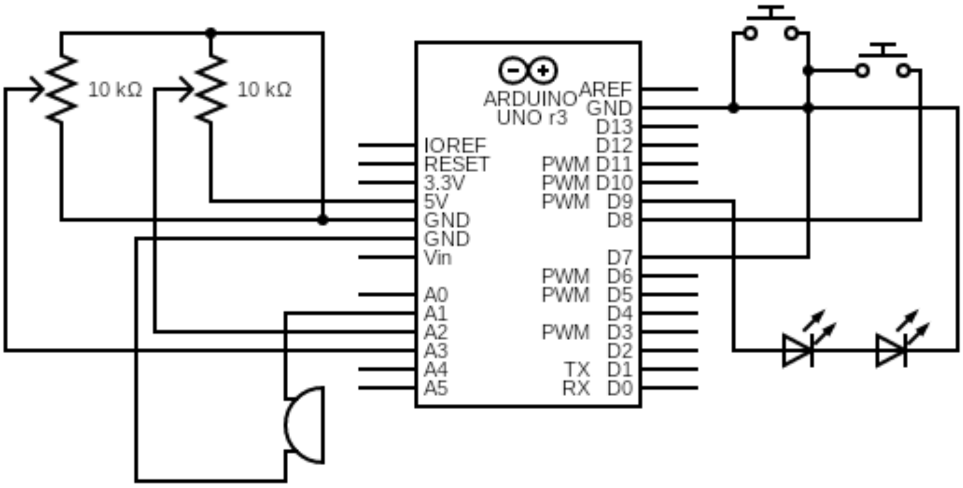

Figure S1: Arudino Uno Rev3 circuit

Videos of individual participants were recorded by GoPro Hero 12 Black cameras mounted on tripods facing the participants. A third GoPro Hero 12 camera recorded both participants' side profiles in the same frame. All cameras were synchronised using the GoPro Quik app to use the same clock and simultaneous recording (start, stop) triggers.

Stimulus presentation and recording

The experiment was implemented in PsychoPy v2023.2.2 stimulus presentation software, using a python package developed specifically for this project. Several steps were taken to ensure precise timing, smooth audiovisual presentation, dynamic logging and recording. Audio playback was handled through the Psychtoolbox audio backend, configured to run in critical latency mode (mode 4) with a dedicated macOS coreaudio driver. MIDI drumming responses were recorded via MAX-MSP v9.0.3, and integrated with PsychoPy using an Open Sound Control (OSC) server running in a dedicated background thread. This allowed uninterrupted and precise recording of incoming MIDI messages, regardless of PsychoPy's on-screen events and pauses in the task flow. Signals for gating sound-sharing, i.e., whether the participants heard each other's drumming or not, were sent via OSC to MAX-MSP depending on the randomised task-sharing blocks. Response devices (buttons and sliders), LEDs and the piezoelectric speaker were controlled via Arduino IDE v2.3.2. These communicated with PsychoPy using the serial v3.5 python package. All media, which included videos, the song track, and audio of the metronomes, were pre-loaded as stimuli at the beginning of the main session to prevent time lags in stimulus presentation. During each trial, the relevant stimuli corresponding to the rhythmic ratio were displayed onto the monitors and played via the headphones. Rating devices were visually presented as custom graphical sliders that dynamically updated marker positions instantaneously depending on the position of the rating slider. MIDI and rating responses were logged trial-by-trial along with relevant metadata to avoid data loss in case of system failure. General timings (e.g., session start, session end) and stimulus-specific event triggers (e.g., metronome start, song start) were logged in each session's log file.

Inferential statistics - IOS Ratings (Q1)

We modelled participants' trial-wise IOS ratings as a function of the interaction of test predictors rhythm type and task-sharing and the standard deviation of interpersonal asynchronies across that trial (henceforth, "combined drumming variability"). We included a combination of control predictors: Trial number accounted for the possible increase in IOS ratings over time. Participant order represented the side bias in the testing room and the assigned part of the rhythmic ratio. Social factors were represented by the initial IOS rating, gender match, song recognition, and B-IRI perspective-taking score. The spontaneous motor tempo difference between the two participants, the overall Gold-MSI score, and the "ability" metric on the BDAT accounted for general musical traits. Random effects included song ID and participant ID, created by concatenating the dyad ID with the participant order. The maximal model including all theoretically identifiable random slopes did not converge. Thus, we removed correlations between random slopes and intercepts to decrease model complexity. Finally, the model converged with the random slope of task-sharing within song ID and participant ID, and participant order within song ID. We considered this our full model. We used a beta distribution with a logit link.

The IOS rating was minimally scaled from a range of 0 to 100 to a range of 0.009804 to 0.990196 so that the response could be handled by the beta model. Combined drumming variability values were log-transformed and z-transformed (scaled to a mean of zero and a standard deviation of 1). All other control covariates (trial number, initial IOS rating, perspective-taking score, Gold-MSI score, and BDAT ability) were also z-transformed to aid model convergence. Random slopes of factors were included as dummy-coded factors to ease interpretation and centered to a mean of zero.

Due to the responses forming a trimodal distribution (Figure S2), the model was mildly underdispersed (dispersion parameter = 0.87). This led to slightly conservative model estimates, increasing the risk of false negatives. VIFs were approximately equal to 1 (max. VIF = 1.035), suggesting no collinearity issues. Plots of BLUPs were approximately normal, within acceptable limits. The ranges of estimates derived from the custom model stability test were fairly narrow and indicated clear directions for the effects of the significant test predictors. Song recognition was the sole exception with wide-ranging estimates. The analysis of the influence of individual participants on the model's estimates yielded a maximum Cook's distance of 0.1093 (Figure S3). Overall, this suggests that our model was highly stable.

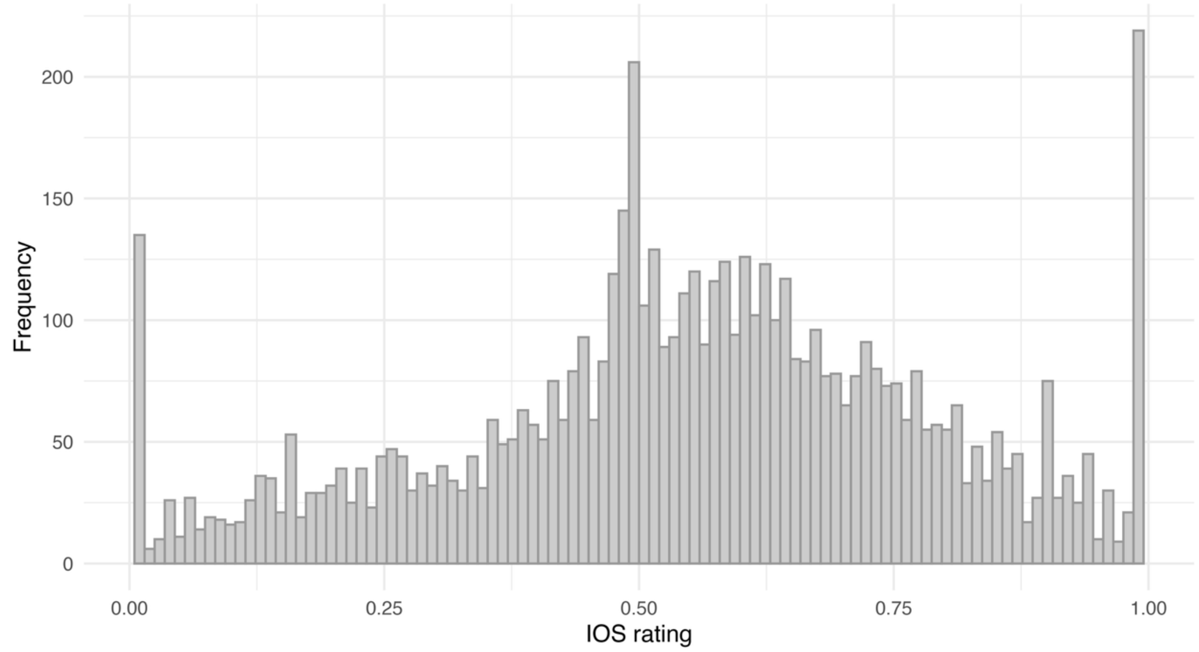

Figure S2: Histogram of IOS ratings

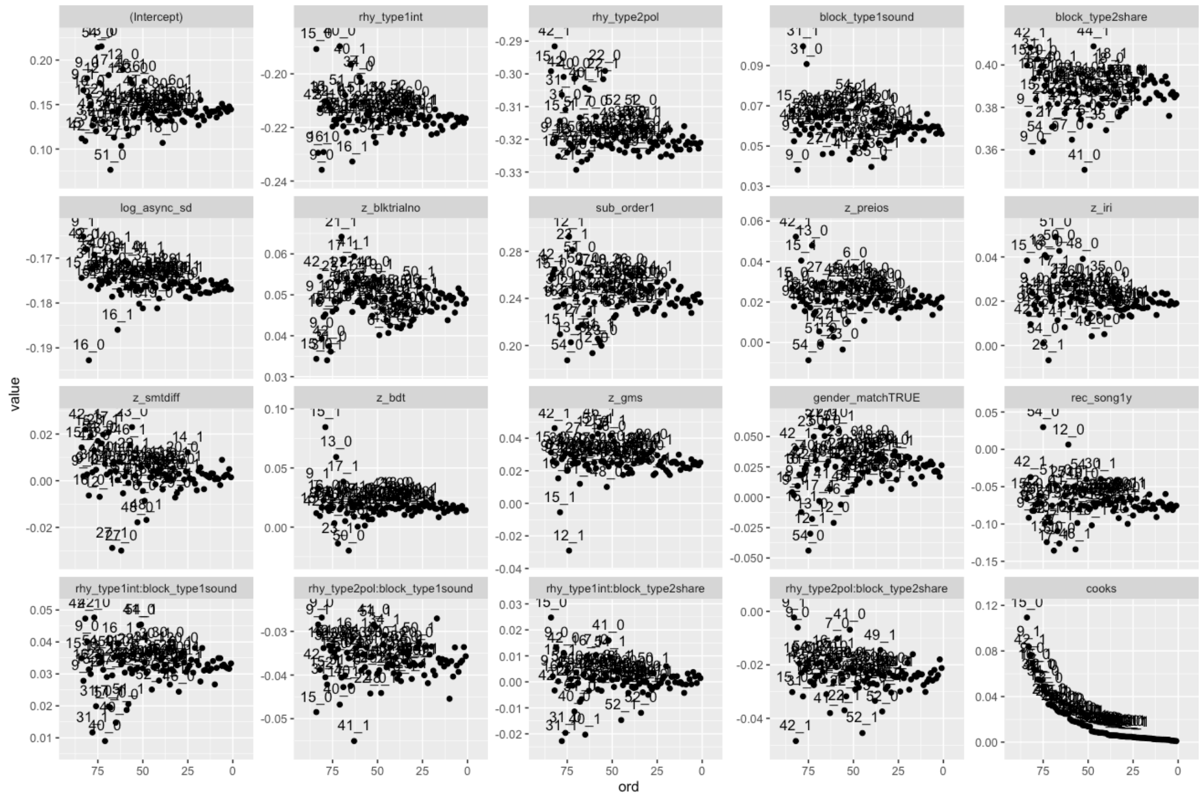

Figure S3: Plot of the influence of removing individual participants on the IOS ratinsgs (Q1) model's estimates of the response for each fixed effects predictor. The final plot (bottom right corner) shows Cook's distance values for each participant ID.

The full-null comparison was significant, but the interaction between rhythm type and task-sharing block was not, therefore, we created a reduced model. This reduced model contained the main effects of rhythm type and task-sharing, but lacked the interaction of the two, and was otherwise identical to the full model. We conducted a post hoc comparison of pairwise estimated marginal means with Tukey p-value adjustment for

the factors rhythm type and task-sharing block. The model had a conditional r-squared of 0.876, and a marginal r-squared of 0.128.

Upon suggestion from a reviewer, we ran an exploratory model testing a possible mediating effect of the combined drumming variability on the effects of the rhythm type and task sharing. To do this, we additionally included a three-way interaction of the three aforementioned variables in a model otherwise identical to our main model of IOS ratings. This model also showed fairly conservative estimates due to underdispersion (dispersion parameter = 0.75). Since the full-null comparison was not significant (p = 0.752), we did not proceed with further testing.

Inferential statistics - Interpersonal Drumming (Q2)

We modelled the effect of one participant's barwise drumming variability (henceforth, "variability") on the other's using a Gaussian GLMM. To do this, we considered participant 1 the response and participant 2 the predictor, and duplicated the dataset to analyse vice versa within the same model. The test predictors were two two-way interaction terms: the real partner's variability with the rhythm type, and the real partner's variability with task-sharing. Since the task setup was such that each dyad experienced very controlled and consistent rhythmic scenarios (background music, metronome cues, etc.), we created pseudo-partners for each participant. This was done by randomly mapping another participant from the study who did not actually perform the task with the participant in question, but experienced the same song with a different partner. The two interaction terms with rhythm type and task-sharing with pseudo-partner variability were, thus, included as control predictors.

Additional control predictors included the trial number, bar number, and participant order. Random effects included dyad ID, participant ID, created by concatenating the dyad ID with the participant order, song ID, and ratio. The maximal model did not converge. Thus, we first removed correlations between random slopes and intercepts to decrease model complexity. This alone did not facilitate convergence, and we finally arrived at a converging full model with the random slopes of rhythm type, task-sharing, and participant order within both dyad ID, and song ID; random slopes of task-sharing and participant order within ratio, and the random slopes of rhythm type and task-sharing within participant ID. Both the response variability and predictor variability were log-transformed and z-transformed. The covariates trial number and bar number were z-transformed. The control factor participant order was dummy coded and centered to include as a random slope.

A dispersion parameter of 0.99 indicated that the model handled the residuals well. VIFs were again close to 1 (max. VIF = 1. 296), and suggested no collinearity issues. Plots of BLUPs were approximately normal, within acceptable limits. The custom stability test showed fairly narrow ranges and clear directions for the effects of the significant test predictors, with the random slope of the polyrhythm within the random effects of dyad ID and participant ID showing wide ranges. Individual dyads did not widely influence the model's estimates indicated by a maximum Cook's distance of 0.5748 (Figure S4). Overall, this suggests that our model was highly stable.

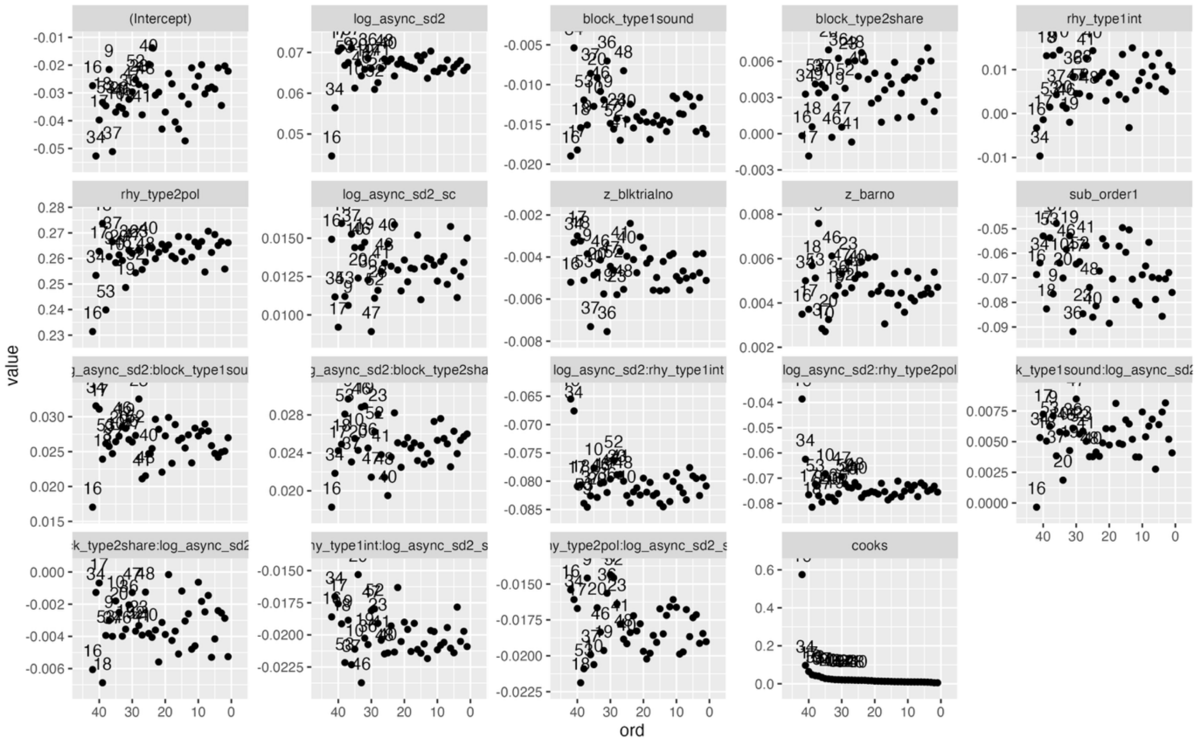

Figure S4: Plot of the influence of removing individual dyads on the interpersonal drumming (Q2) model's estimates of the response for each fixed effects predictor. The final plot (bottom right corner) shows Cook's distance values for each dyad ID.

Post hoc comparisons were conducted using estimated marginal trends (simple slopes) with Holm correction, allowing pairwise contrasts of the effect of the continuous predictor partner variability across factor levels within rhythm type and task-sharing. We obtained a conditional r-squared of 0.209, and a marginal r-squared of 0.014.

Our hypotheses warranted an exploratory analysis of two three-way interactions of partner variability, rhythm type and task-sharing block and pseudo-partner variability, rhythm type and task-sharing block. To do this, we ran a model identical to the main analysis, but with both three-way interactions. This model resulted in a singular fit. Therefore, the random effects structure had to be simplified to include only one random effect of dyad ID with random slopes for rhythm type, task-sharing, and participant order. This model also showed somewhat conservative estimates (dispersion parameter = 0.88). We limited further analysis for this model to likelihood ratio tests of the fixed effects terms (including the three-way interaction), and 1000 parametric bootstraps to obtain 95% confidence intervals for the key significant test predictors.
