# Supplementary material for: Rhythmic Structure Shapes Dyadic Self−Other Representations Through Interpersonal Action Coupling
Source: Ann N Y Acad Sci. 2026 Aug 3;1562(1):e70344. doi: 10.1111/nyas.70344 (PMC13432789; doi:10.1111/nyas.70344)
Supplement: Supplementary file 6 — Supplementary Information: nyas70344‐sup‐0006‐Figures_S5‐S7.pdf. [file NYAS-1562-0-s007.pdf]

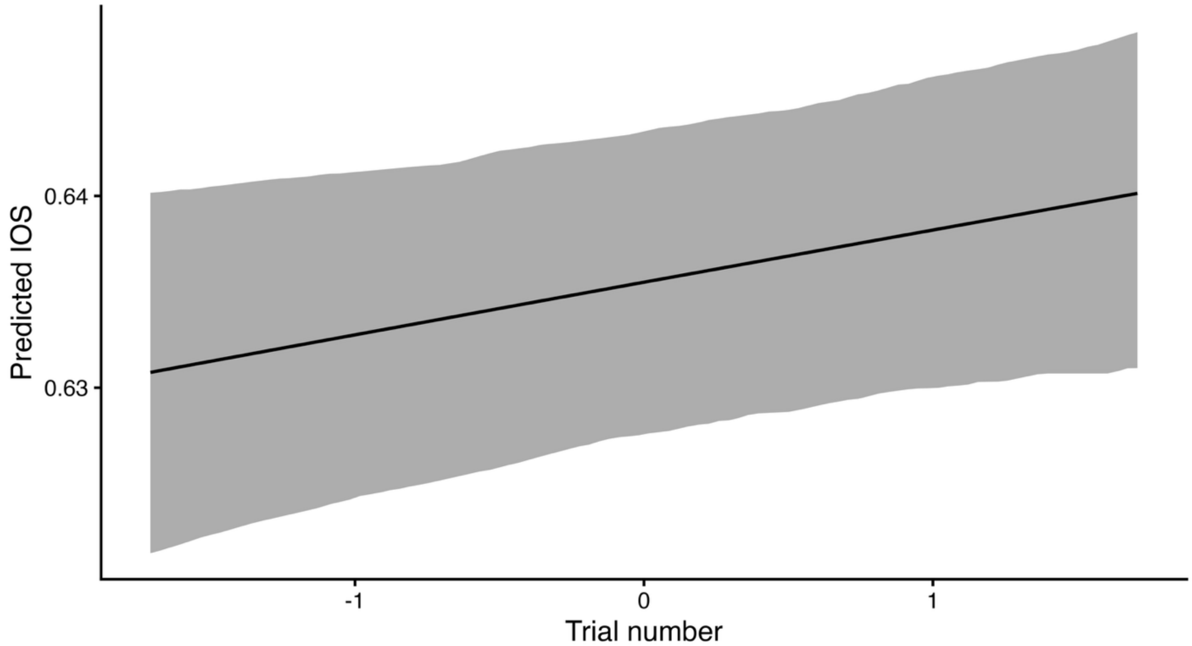

Figure S5: Bootstrapped model fitted estimates (Q1) of IOS ratings and 95% confidence intervals for the main effect of trial number ($p < 0.05$; x-axis depicts z-transformed values), conditional on the effects of all other covariates and factors in the model being zero.

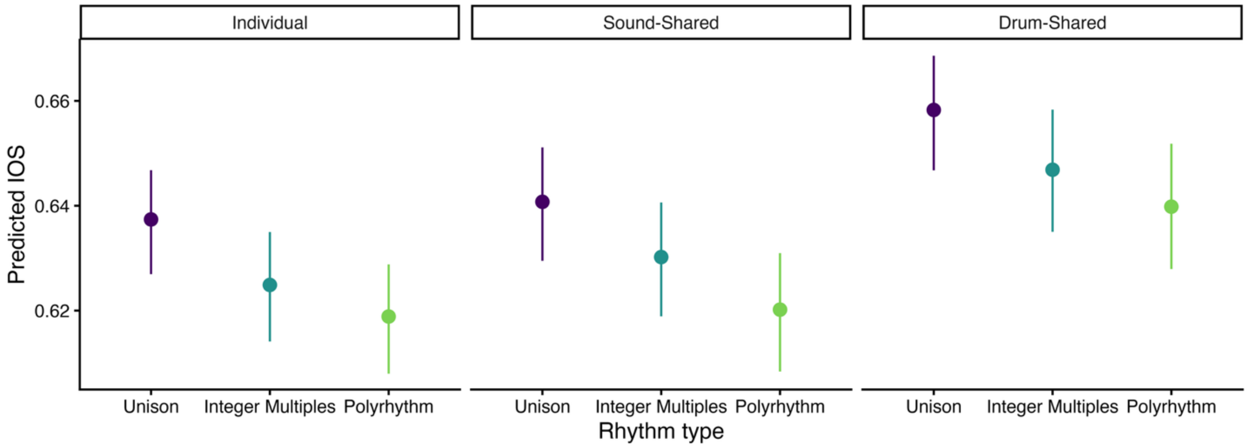

Figure S6: Bootstrapped model fitted estimates (Q1) of IOS ratings and 95% confidence intervals for the non-significant test interaction of rhythm type and task-sharing (p = 0.86), conditional on the effects of all other covariates and factors in the model being zero.

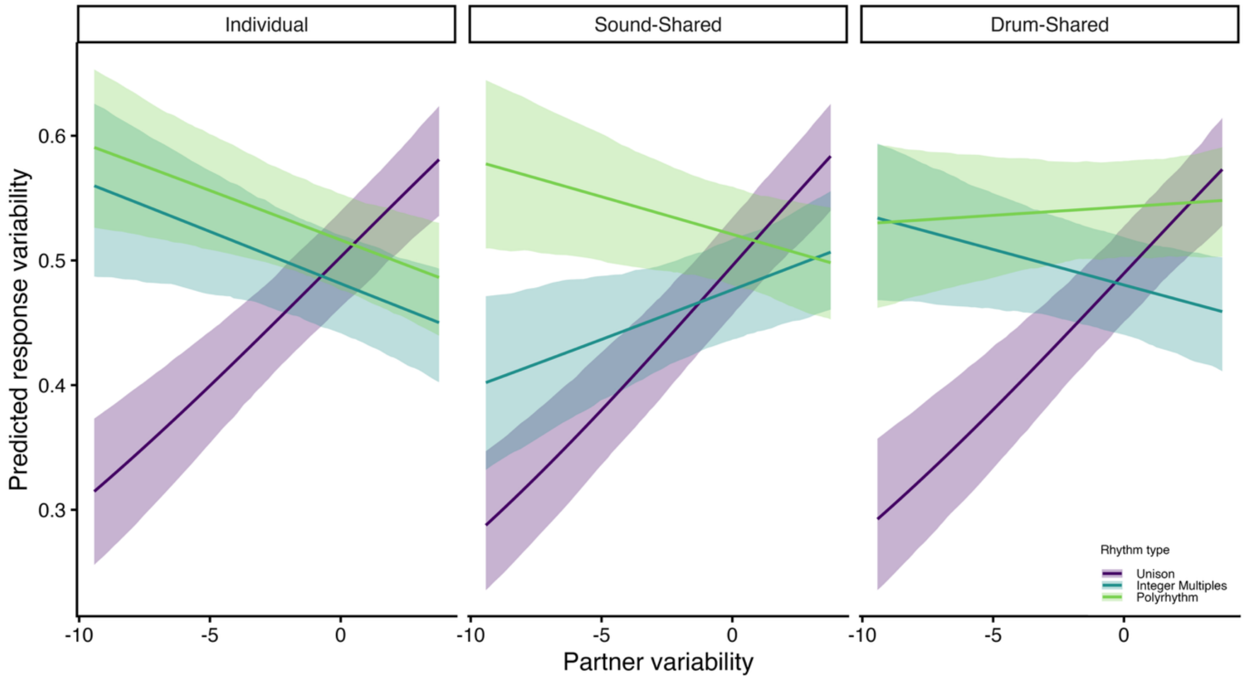

Figure S7: Bootstrapped model fitted estimates (Q2) of response variability (lines) and 95% confidence intervals (shaded bands) for the three-way interaction of log-transformed of partner variability with task-sharing block, and rhythm type ($p < 0.001$), conditional on the effects of all other covariates and factors in the model being zero. Estimates and confidence intervals are from a model with the 3-way test interaction partner variability × rhythm type × task-sharing.
