# Supplementary material for: Rhythmic Structure Shapes Dyadic Self−Other Representations Through Interpersonal Action Coupling
Source: Ann N Y Acad Sci. 2026 Aug 3;1562(1):e70344. doi: 10.1111/nyas.70344 (PMC13432789; doi:10.1111/nyas.70344)
Supplement: Supplementary file 7 — Supplementary Information: nyas70344‐sup‐0007‐Tables_S1‐S5.pdf. [file NYAS-1562-0-s005.pdf]

Table S1: Summary R packages and functions used for inferential statistics. Symbols: none - used in both IOS and interpersonal drumming analyses, † - used in IOS (Q1) analyses, ˜ - used in interpersonal drumming (Q2) analyses.

Purpose	Function	Arguments	Package	Version
Generalised linear mixed model definition	glmmTMB	†family = beta_family(link = "logit")<br>˜family = gaussian(link = "identity")	glmmTMB	1.1.13
Data exploration to define maximal random effects structure	fe.re.tab	†use = c("rhythm_type", "task_sharing", "interpersonal_variability)<br>˜use = c(("rhythm_type", "task_sharing", "partner_variability)	Mundry, 2023	
Parametric bootstrap, 95% confidence intervals	boot.glmmtmb		Mundry, 2023	
Overdispersion test	testDispersion	type = "DHARMa",<br>alternative = "two.sided"	DHARMa	0.4.7
Model definition for collinearity test	lm		base	4.4.0
Collinearity test using Variance Inflation Factors	vif		car	3.1.2
Model stability - Overall	glmmTMB.stab (wrapper of glmmTMB.stability)		Mundry, 2023	
Model stability – Influence of random effects	influence_mixed, cooks.distance	†groups = "participant_id"<br>˜groups = "dyad_id"	glmmTMB, stats	
Full-null model comparison	anova	test = "Chisq"	base	4.4.0
Individual effects of predictors	drop1	test = "Chisq"	base	4.4.0
Effect size estimation	r2		performance	0.15.2
Post hoc pairwise contrasts	emmeans, pairs	†adjust = "tukey"	emmeans	1.10.2
Post hoc estimated marginal trends	emtrends, pairs	˜adjust = "holm"	emmeans	1.10.2

Table S2: Model summary (Q1) in link space for response variable IOS and the main effects and main effects (no interaction - reduced model) of key test predictors rhythm type and task-sharing, and the main effect of log-transformed combined drumming variability.

Explanatory variables	Estimate	Standard Error	z value	p value	
Intercept	0.14554	0.145147	1.003	0.31601	
Rhythm type Integer multiples[1]	-0.205661	0.02565	-8.018	1.07E-15	***
Rhythm type Polyrhythm[1]	-0.338844	0.026025	-13.02	<2.00E-16	***
Task-sharing Sound-shared[1]	0.057433	0.066205	0.868	0.38566	
Task-sharing Drum-shared[1]	0.377801	0.119214	3.169	0.00153	**
Log Drumming variability[2]	-0.176689	0.014809	-11.931	<2.00E-16	***
Trial number[2]	0.047841	0.022507	2.126	0.03353	*
Participant order P2[1]	0.239964	0.142996	1.678	0.09332	.
Initial IOS rating2	0.019923	0.072414	0.275	0.78322	
B-IRI Perspective-taking[2]	0.019772	0.071893	0.275	0.7833	
Spontaneous motor tempo difference[2]	0.002244	0.072986	0.031	0.97547	
B-DAT Ability[2]	0.016144	0.075201	0.215	0.83002	
Gold-MSI score[2]	0.024628	0.074829	0.329	0.74206	
Gender match True[1]	0.022931	0.143981	0.159	0.87346	
Song recognition True[1]	-0.07465	0.167653	-0.445	0.65613	

Significance codes: . < 0.1, * < 0.05, ** < 0.01, *** < 0.001

1 Reference levels: “Unison” for rhythm type, “Individual” for task-sharing, “P1” for participant order, “False” for gender match, “False” for song recognition

2 z-transformed to an approximate mean of zero and sd of 1

Table S3: IOS ratings (Q1) - random effects variances for the full model

Grouping factor	Effect[1]	SD
Participant ID	(Intercept)	0.66
	Task-sharing Sound-shared	0.98
	Task-sharing Drum-shared	1.22
Song ID	(Intercept)	0.26
	Task-sharing Sound-shared	0.26
	Task-sharing Drum-shared	2.52E-05
	Participant order P2	0.49

[1] "intercept" depicts a random intercept, all others random slopes

Table S4: Interpersonal drumming (Q2) - random effects variances for the full model

Grouping factor	Effect[1]	SD
Dyad ID	(Intercept)	0.21143
	Task-sharing Sound-shared	0.05800
	Task-sharing Drum-shared	0.00003
	Rhythm type Integer multiples	0.12984
	Rhythm type Polyrhythm	0.20626
	Participant order P2	0.00034
Participant ID	(Intercept)	0.34625
	Task-sharing Sound-shared	0.08529
	Task-sharing Drum-shared	0.11288
	Rhythm type Integer multiples	0.13635
	Rhythm type Polyrhythm	0.19329
Ratio	(Intercept)	0.08274
	Task-sharing Sound-shared	0.00625
	Task-sharing Drum-shared	0.06746
	Participant order P2	0.16039
Song ID	(Intercept)	0.00006
	Task-sharing Sound-shared	0.00001
	Task-sharing Drum-shared	0.00001
	Rhythm type Integer multiples	0.07997
	Rhythm type Polyrhythm	0.00003
	Participant order P2	0.00009
Residual		0.88593

[1] "intercept" depicts a random intercept, all others random slopes

Table S5: Model summary (Q2) for log-transformed response variability and the 3-way interaction of key test predictors rhythm type, task-sharing, and log-transformed partner variability

Explanatory variables	Estimate	Standard Error	z value	p value	
Intercept	0.06234	0.0683	0.91	0.36137	
Log Partner variability[2]	0.08381	0.0122	6.87	6.60E-12	***
Task-sharing Sound-shared[1]	-0.02864	0.02099	-1.36	0.17239	
Task-sharing Drum-shared[1]	-0.05307	0.01905	-2.78	0.00535	**
Rhythm type Integer multiples[1]	-0.08587	0.03271	-2.63	0.00865	**
Rhythm type Polyrhythm[1]	0.05409	0.04268	1.27	0.20506	
Log Pseudopartner variability[2]	0.03515	0.01217	2.89	0.00388	**
Trial number[2]	-0.00584	0.00582	-1	0.31519	
Bar number[2]	0.00472	0.0039	1.21	0.22523	
Participant order[1]	-0.10156	0.07459	-1.36	0.17332	
Log Partner variability : Sound-shared	0.01063	0.01713	0.62	0.53492	
Log Partner variability : Drum-shared	0.00552	0.01746	0.32	0.75182	
Log Partner variability : Integer multiples	-0.11723	0.01727	-6.79	1.10E-11	***
Log Partner variability : Polyrhythm	-0.11576	0.01694	-6.84	8.20E-12	***
Sound-shared : Integer multiples	0.00889	0.0233	0.38	0.70267	
Drum-shared : Integer multiples	0.04869	0.02358	2.06	0.03894	*
Sound-shared : Polyrhythm	0.04694	0.02328	2.02	0.04375	*
Drum-shared : Polyrhythm	0.15981	0.02363	6.76	1.40E-11	***
Sound-shared : Log Pseudopartner variability	-0.0101	0.01711	-0.59	0.55498	
Drum-shared : Log Pseudopartner variability	-0.03306	0.01735	-1.91	0.05674	.
Integer multiples : Log Pseudopartner variability	-0.06092	0.01715	-3.55	0.00038	***
Polyrhythm : Log Pseudopartner variability	-0.04063	0.01687	-2.41	0.016	*
Log Partner variability : Sound-shared : Integer multiples	0.05493	0.0239	2.3	0.02155	*
Log Partner variability : Drum-shared : Integer multiples	0.00511	0.02403	0.21	0.83172	
Log Partner variability : Sound-shared : Polyrhythm	-0.00292	0.02363	-0.12	0.90151	
Log Partner variability : Drum-shared : Polyrhythm	0.0319	0.02367	1.35	0.17765	
Sound-sh. : Integer mult. : Log Pseudopartner variability	0.03684	0.02394	1.54	0.12396	
Drum-sh. : Integer mult. : Log Pseudopartner variability	0.04429	0.02398	1.85	0.06476	.
Sound-shared : Polyrhythm : Log Pseudopartner variability	0.00111	0.02361	0.05	0.96259	
Drum-shared : Polyrhythm : Log Pseudopartner variability	0.02763	0.02358	1.17	0.24142	

Significance codes: . < 0.1, * < 0.05, ** < 0.01, *** < 0.001
[1] Reference levels: “Unison” for rhythm type, “Individual” for task-sharing, “P1” for participant order, “False” for gender match, “False” for song recognition
[2] z-transformed to an approximate mean of zero and sd of 1
